# A comparison of healing and complication rates between common flaps utilized in total knee arthroplasty: a review of the literature

**DOI:** 10.1186/s43019-022-00145-3

**Published:** 2022-03-26

**Authors:** Akhil A. Chandra, Filippo Romanelli, Alex Tang, Luke Menken, Maximilian Zhang, Adam Feintisch, Frank A. Liporace, Richard S. Yoon

**Affiliations:** 1 Rutgers Robert Wood Johnson Medical School, 675 Hoes Lane West, Piscataway, NJ 08854 USA; 2 Division of Orthopaedic Trauma and Adult Reconstruction, Department of Orthopaedic Surgery, Jersey City Medical Center – RWJ Barnabas Health, 355 Grand Street, Jersey City, NJ 07302 USA; 3 Division of Orthopaedic Trauma and Adult Reconstruction, Department of Orthopaedic Surgery, Jersey City Medical Center – RWJBarnabas Health, 377 Jersey Ave, Suite 280A, Jersey City, NJ 07302 USA

**Keywords:** Knee, Arthroplasty, Revision, Flap

## Abstract

**Background:**

Flap reconstruction with perforator, fasciocutaneous, muscular, and/or free microvascular flaps is utilized to cover wound defects and improve vascularization and antibiotic/nutrient delivery. Flap use in revision procedures for total knee arthroplasty has been explored previously; however, current data are limited and studies comparing healing and complication rates between different flap types are lacking.

**Methods:**

A literature review was performed using PubMed on 13 January 2022. Studies were included if they reported healing and complication rates for either gastrocnemius, rectus abdominis, latissimus dorsi, fasciocutaneous, chimeric, or gracilis flaps in the setting of revision total knee arthroplasty (TKA).

**Results:**

The final cohort included gastrocnemius (*n* = 421, healing rate 73.8%, complication rate 59.9%), gracilis (*n* = 9, healing rate 93%, complication rate 55.6%), latissimus dorsi (*n* = 41, healing rate 67%, complication rate 46.3%), rectus abdominis (*n* = 3, healing rate 100%, complication rate 0%), fasciocutaneous (*n* = 78, healing rate 70%, complication rate 19.2%), and chimeric flaps (*n* = 4, healing rate 100%, complication rate 25%). There was no significant difference when comparing healing rates across flap types (*p* = 0.39). There was a significant difference when comparing complication rates across flap types (*p* < 0.0001), with a significant difference being noted between gastrocnemius and fasciocutaneous complication rates (*p* < 0.0001). All other comparisons between flap types by complication rate were not significantly different.

**Conclusions:**

Gastrocnemius flaps are the workhorse flap in the setting of revision TKA, as evidenced by this review. Healing rates did not vary significantly across flap types, which suggests that determining the appropriate flap for coverage of soft-tissue defects in revision TKA should be driven by defect size and location as well as physician experience and patient tolerance.

## Background

Total knee arthroplasty (TKA) provides excellent results for most patients, with literature estimating revision rates less than 5.0% over 10 years [1]. However, complications from TKA revision surgeries are high and include wound dehiscence, infection, aseptic loosening, and, in cases where the limb cannot be salvaged, above-knee amputation (in 0.1% of cases) [2, 3]. An exposed prosthetic implant after TKA poses substantial risk to patient morbidity and mortality. In the setting of either septic or aseptic soft tissue loss, flap reconstruction is a viable treatment option.

Thus, the indications for flap use in the setting of revision TKA include but are not limited to infection [4], implant exposure [5], disruption of the extensor mechanism (from patellar tendon tear/avulsion, patellar fracture, or quadriceps tendon tear) [6], soft-tissue coverage [7], and limb salvage [8]. In particular, the flap pattern provides a vascular supply for an overlying skin graft, which improves oxygen and nutrient delivery to the overlying skin graft and the underlying anatomic structures. Further, improved blood supply facilitates dissemination of systemic antibiotics to the surgical site, thereby decreasing nidus for future infections.

Flap use in revision procedures for TKA has been explored frequently, with various studies commenting on the operative technique and effectiveness. However, the current data reported on flap use in revision TKA are mainly outdated and limited to case reports, case series, technical tricks, and small sample size (Tables 1, 2, 3, 4, 5, 6, 7). Furthermore, studies comparing healing rates between different flap types are lacking [7, 9]. Therefore, the aim of this review is to provide a concise review of the current literature on this topic and to assess the healing and complication rates for each commonly used flap type in the setting of revision TKA.

**Table 1:** Flap type by success and complication rate

Flap type	Sample size (*n*)	Healing rate (%)	Complication rate (%)
Gastrocnemius	421	311 (73.8)	252 (59.9)
Rectus abdominis	3	3 (100.0)	0 (0.0)
Latissimus dorsi	41	27 (67.0)	19 (46.3)
Fasciocutaneous	78	55 (70.0)	15 (19.2)
Chimeric	4	4 (100.0)	1 (25.0)
Gracilis	9	8 (93.0)	5 (55.6)

**Table 2:** Gastrocnemius

Author	Flap type	Healing rate	Complications	Mean f/u (months)	Minimum f/u (months)	Maximum f/u (months)
Adam et al.	Medial gastrocnemius (*n* = 14)	57.1%	Infection (*n* = 14)	64.8	12	120
Anract et al.	Gastrocnemius + pes anserinus (*n* = 4) Gastrocnemius (*n* = 5)	100%	Infection and arthrodesis (*n* = 1)	23	6	30
Boopalan et al.	Medial + lateral gastrocnemius (*n* = 1)	100%	None	18	–	–
Busfield et al.	Medial gastrocnemius (*n* = 9)	71.4%	Wound breakdown (*n* = 1) Infection (*n* = 1)	21	7	31
Carlesimo et al.	Medial gastrocnemius (*n* = 8)	100%	None	21.6	8	36
Casanova et al.	Medial gastrocnemius (*n* = 4) Lateral gastrocnemius (*n* = 2) Medial + lateral gastrocnemius (*n* = 1)	85.7%	Infection (*n* = 1)	28	14	59
Chiou et al.	Lateral gastrocnemius–Achilles tendon (*n* = 1)	100%	None	18	–	–
Corten et al.	Gastrocnemius (*n* = 24)	92%	Nonhealing (*n* = 2) Wound breakdown (*n* = 1) Infection (*n* = 1) Above-knee amputation (*n* = 1)	54	12	120
Gerwin et al.	Medial gastrocnemius (*n* = 12)	92%	Wound necrosis (*n* = 1)	41	19	119
Houdek et al.	Gastrocnemius (*n* = 83)	68%	At least one postoperative complication (*n* = 59) Multiple complications (*n* = 32)	96	24	240
Jaureguito et al.	Medial gastrocnemius (*n* = 6)	100%	Manipulation under anesthesia (*n* = 1) Infection (*n* = 1)	33	26	41
Markovich et al.	Medial gastrocnemius (*n* = 6)	100%	None	49.2	12	96
McPherson et al.	Medial gastrocnemius (*n* = 21)	95.2%	Skin necrosis (*n* = 4) Partial necrosis skin graft (*n* = 3) Partial peroneal nerve palsy (*n* = 3) Flare of preexisting reflex sympathetic dystrophy (*n* = 3) Chronic lower leg swelling (*n* = 3) Intraoperative fracture of femoral condyle (*n* = 2) Patellar tendon rupture (*n* = 2) Patellar tendon stretch (*n* = 1) S1 nerve palsy (*n* = 1) Knee stiffness (*n* = 1) Recurrent knee infection (*n* = 1) Catheter infection (*n* = 2) Fat embolus (*n* = 1) Cholecystitis requiring cholecystectomy (*n* = 1)	17	5.1	33.1
Moog et al.	Medial gastrocnemius (*n* = 20)	75%	Nonhealing (*n* = 3) Infection (*n* = 5)	24.2 ± 3.1	–	–
Nahabedian et al.	Gastrocnemius (*n* = 19)	79%	Partial necrosis (*n* = 3) Nonhealing (*n* = 1)	70.7	24	120
Ng et al.	Medial gastrocnemius (*n* = 1)	0%	Dehiscence (*n* = 1)	24	24	24
Pozzobon et al.	Medial gastrocnemius (*n* = 8)	89%	Amputation (*n* = 4)	25.2	5	60
Ries et al.	Medial gastrocnemius (*n* = 12)	92%	Additional coverage (*n* = 3) Above-knee amputation (*n* = 1)	28	18	48
Siim et al.	Gastrocnemius (*n* = 4)	25%	Fistula (*n* = 2) Poor vascularization (*n* = 1)	96	12	204
Suda et al.	Lateral gastrocnemius (*n* = 2) Medial gastrocnemius (*n* = 12) Lateral + medial gastrocnemius (*n* = 1)	47%	Lateral gastrocnemius: Minor complication (*n* = 1) Medial gastrocnemius: Minor complications (*n* = 3) Major complications (*n* = 2) Infection (*n* = 2)	37	13	61
Tetreault et al. (2017)	Medial gastrocnemius (*n* = 31)	48%	Infection (*n* = 14)	48	24	72
Tetreault et al. (2016)	Medial gastrocnemius (*n* = 27)	48%	Infection (*n* = 14)	48	24	72
Theil et al.	Lateral gastrocnemius (*n* = 6) Medial gastrocnemius (*n* = 37)	65%	Infection (*n* = 15)	53 (median)	18	79
Warren et al.	Medial gastrocnemius (*n* = 24) Lateral gastrocnemius (*n* = 1)	42%	Lateral gastrocnemius: Arthrodesis (*n* = 1) Medial gastrocnemius: Recurrent infection (*n* = 18) Arthrodesis (*n* = 4) Above-knee amputation (*n* = 5)	39.6	1.2	216
Young et al.	Gastrocnemius (*n* = 15)	73%	Above-knee amputation (*n* = 3) Arthrodesis (*n* = 1)	33	5	96

**Table 3:** Rectus abdominis

Author	Flap type	Healing rate	Complications	Mean f/u (months)	Minimum f/u (months)	Maximum f/u (months)
Browne et al.	Rectus abdominis (*n* = 1)	100%	None	24	–	–
Markovich et al.	Rectus abdominis (*n* = 2)	100%	None	49.2	12	96

*f/u* follow-up

**Table 4:** Latissimus dorsi

Author	Flap type	Healing rate	Complications	Mean f/u (months)	Minimum f/u (months)	Maximum f/u (months)
Adam et al.	Latissimus dorsi (*n* = 2)	100%	Nonhealing (*n* = 1)	64.8	12	120
Hierner et al.	Latissimus dorsi (*n* = 16)	50%	Infection (*n* = 3) Stiffness/arthrolysis (*n* = 1)	34.6	12	59
Markovich et al.	Latissimus dorsi (*n* = 5)	40%	Recurrent infection (*n* = 3)	49.2	12	96
Raymond et al.	Latissimus dorsi (*n* = 18)	77.8%	Long-term antibiotics (*n* = 7) Amputation (*n* = 4)	49	18	110

**Table 5:** Fasciocutaneous

Author	Flap type	Healing rate	Complications	Mean f/u (months)	Minimum f/u (months)	Maximum f/u (months)
Adam et al.	Saphenous fasciocutaneous (*n* = 5) Fasciocutaneous posterior calf (*n* = 3)	100%	None	64.8	12	120
Menderes et al.	Fasciocutaneous (*n* = 7)	100%	Revision (*n* = 3)	20	1	61
Misra et al.	Fasciocutaneous (*n* = 15)	100%	Wound dehiscence (*n* = 1)	13	3	24
Nahabedian et al.	Fasciocutaneous (*n* = 5)	80%	Nonhealing (*n* = 1)	81.6	48	120
Papaioannou et al.	Fasciocutaneous (*n* = 16)	94	Partial flap loss (*n* = 1)	28	6	60
Siim et al.	Fasciocutaneous (*n* = 10)	60%	Infection (*n* = 1) Partial vascularization (*n* = 1) Partial necrosis (*n* = 2)	57.6	12	156
Suda et al.	Fasciocutaneous (*n* = 1)	0%	Above-knee amputation (*n* = 1)	37	13	61
Vaienti et al.	Island sural neurocutaneous flap (*n* = 15)	100%	Hematoma (*n* = 2) Aseptic fistula (*n* = 1)	18	–	–
Young et al.	Fasciocutaneous (*n* = 1)	0%	Above-knee amputation (*n* = 1)	33	5	96

**Table 6:** Chimeric

Author	Flap type	Healing rate	Complications	Mean f/u (months)	Minimum f/u (months)	Maximum f/u (months)
Cho et al.	Medial gastrocnemius and medial sural artery adipofascial (*n* = 1)	100%	None	36	–	–
Fu et al.	Chimeric ALT perforator flap with fascia lata (*n* = 1)	100%	None	16.5	8	35
Hallock et al.	Gastrocnemius muscle–chimeric sural artery perforator flap (*n* = 2)	100%	Revision (*n* = 1)	13	12	14

**Table 7:** Gracilis flap

Author	Flap type	Healing rate	Complications	Mean f/u (months)	Minimum f/u (months)	Maximum f/u (months)
Jung et al.	Gracilis (*n* = 1)	100%	None	24	–	–
Mitsala et al.	Gracilis (*n* = 5)	80%	Infection (*n* = 3) Hematoma (*n* = 2)	25	8	60
Tiengo et al.	Reversed gracilis (*n* = 3)	100%	None	23.3	22	24

## Approach for flap reconstruction

**Fig. 1:**
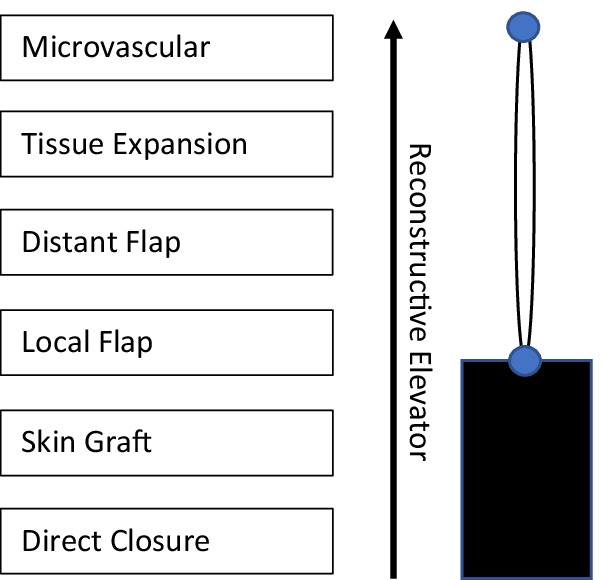
Reconstructive elevator. This methodology encourages surgeons to pick the most appropriate flap type as opposed to addressing flap reconstruction in a sequential, least-complex method. Adapted from Gottlieb et al. (Gottlieb LJ, Krieger LM (1994). From the reconstructive ladder to the reconstructive elevator. *Plast Reconstr Surg* 93:1503–1504)

The current accepted version of the reconstructive ladder was proposed by Gottlieb et al. [10], who proposed the concept of a “reconstructive elevator” as opposed to the “reconstructive ladder,” the main difference being that clinicians should opt for the most appropriate flap rather than the least complex [11] (Fig. 1). In the traditional model, to utilize a local and/or distant flap, clinicians were first advised to begin with primary closure followed by a skin graft. To attempt a more directed approach, especially in the setting of TKA, local (or rotational) and distant (or free) flaps may be attempted directly. Some primary considerations for flap choice include donor site morbidity and defect size. In the setting of TKA, while gastrocnemius flaps have been well described as the main workhorse flap, studies have suggested that superior geniculate artery perforator flaps in place of gastrocnemius flaps or anterolateral thigh flaps in place of rectus or latissimus dorsi flaps may result in less donor site morbidity [11]. It is the aim of this review that, by presenting updated data on healing and complication rates for various flap types, surgeons may be better informed when deciding on flap reconstruction techniques. Institutional review board approval was not required for this review.

## Methods

### Literature search strategy

The methodology utilized in this systematic review follows the Preferred Reporting Items for Systematic Reviews and Meta-Analyses (PRISMA) guidelines. A comprehensive search of the PubMed electronic database was conducted on 13 January 2022. The following keywords were entered into the search engine: ("Surgical Flaps"[Mesh] OR (muscle flaps) OR flaps[tiab] OR "surgical flaps"[tiab] OR Gastrocnemius [tiab] OR "Anterolateral Thigh"[tiab] OR "Rectus Abdominis"[tiab] OR "Rectus Abdominis"[Mesh] OR "Latissimus Dorsi"[tiab] OR "Superficial Back Muscles"[Mesh] OR Fasciocutaneous[tiab] OR Chimeric[tiab] OR "Chimera"[Mesh] OR Gracilis[tiab] OR "Gracilis Muscle"[Mesh]) **AND** ("Arthroplasty, Replacement, Knee"[Mesh] OR ("Reconstructive Surgical Procedures"[Mesh] AND "Knee"[Mesh]) OR (total knee arthroplast*) OR TKA OR (total knee replacement) OR (revision total knee arthroplast*) OR (revision total knee replacement)).

### Eligibility criteria

To meet inclusion criteria, articles needed to be (1) published in the English language, (2) have full-text information readily available online, (3) report flap coverage to treat exposed primary total knee arthroplasty (TKA) following infection using either gastrocnemius, rectus abdominis, latissimus dorsi, fasciocutaneous, chimeric, or gracilis flaps, (4) report clinical outcomes that include complications and survival or wound healing rates related to the flap, and (5) have a minimum follow-up of at least 1 year. Articles were excluded from our final analysis if they (1) did not meet inclusion criteria, (2) studied flap coverage in revision surgeries unrelated to an exposed TKA (e.g., arthrodesis, trauma of the native joint), (3) were in vitro studies (including biomechanical studies), review articles, systematic reviews, or letters to the editor, (3) reported nonclinical outcomes, or (4) reported treatment on any joint that is not the knee.

### Study selection

**Fig. 2:**
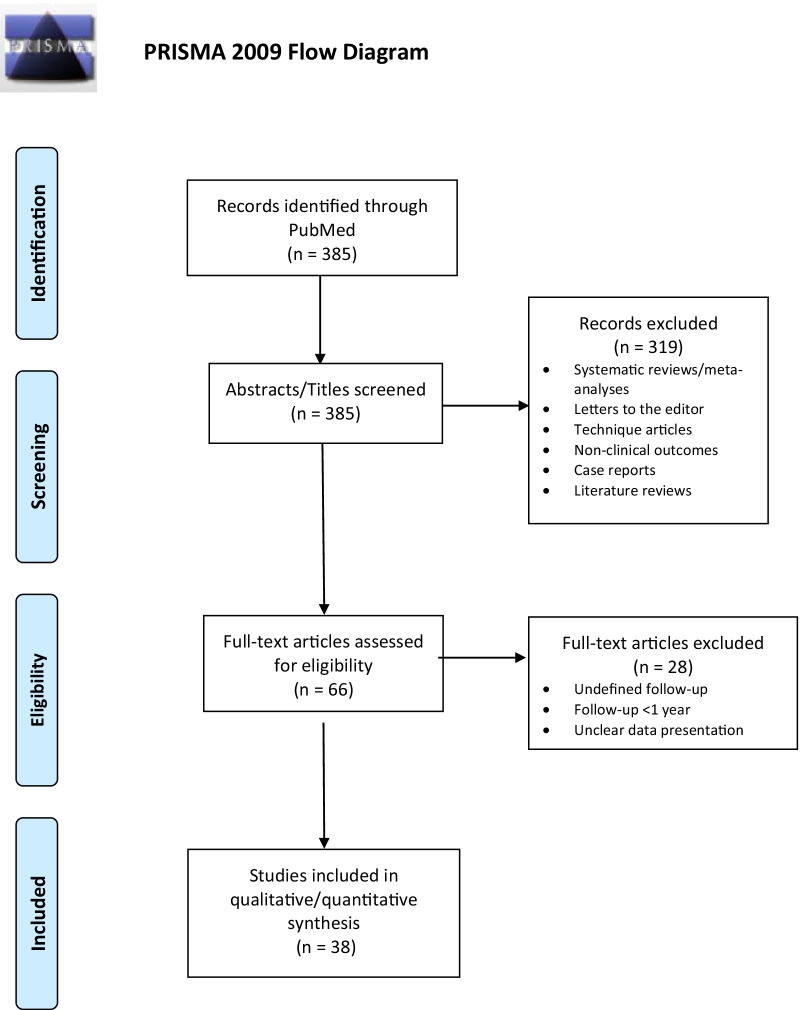
Literature search consort diagram

The initial systematic search generated a total of 385 studies. Two reviewers (A.A.C. and A.T.) independently screened each article for eligibility, extracted data on a separate spreadsheet, and evaluated the methodology and level of evidence for quality assurance. Any disagreement between the two reviewers was resolved through consultation with the senior author (R.S.Y.). Out of the 385 studies identified, only 66 studies were included for full-text review. Of the 66 full-text reviews, 28 studies were excluded while 38 studies were included for qualitative/quantitative analysis (Fig. 2).

### Data extraction

Patients were then divided into different groups based on flap type: gastrocnemius, rectus abdominis, latissimus dorsi, fasciocutaneous, chimeric, or gracilis flaps. The primary outcome was flap survival/wound healing rates and complications compared between the different groups (Table 1).

### Statistical analysis

Descriptive data are reported as counts (%). To compare data between each groups, continuous variables (i.e., demographic data) were analyzed using independent *t*-tests for parametric variables and Mann–Whitney *U* test for nonparametric variables, while categorical variables (i.e., wound healing and complications data) were analyzed using chi-squared tests. Statistical significance was set at *p* ≤ 0.05. All statistical analyses were performed using SPSS version 25 (IBM Corporation, Armonk, New York).

### Flap introduction and operative anatomy

#### Pedicled/local flaps

##### Gastrocnemius

The gastrocnemius muscle’s medial head originates from the posterior medial femoral condyle, while the lateral head extends from the posterior lateral femoral condyle; the two parts combine distal to the myotendinous junction and form an aponeurosis, which combines with the aponeurosis of the soleus muscle to become the Achilles tendon [12]. The gastrocnemius is supplied by the sural arteries (downstream of the popliteal artery) and is drained via the popliteal vein; the muscle mainly aids in plantar flexion and supination of the foot by coordination from the tibial nerve [13].

Walton et al. described the operative approach for completing a lateral gastrocnemius muscle flap [14]. In their description, the first incision is made 2 cm posterior to and parallel to the fibula beginning at the level of the fibular head and extending 10 cm above the distal fibula. Distally, the muscle is followed until the Achilles tendon and carefully resected; a portion of the Achilles tendon may also be taken to aid in reconstruction. The lateral flap is then rotated anteriorly over the fibula to cover soft-tissue defects.

For a medial gastrocnemius flap, an incision may be made from the medial tibia (posterior to the pes anserine tendons) and extended until the superficial posterior compartment until about 10 cm superior to the ankle; in particular, the sural artery should be identified early [14]. The neurovascular structures in the posterior compartment must be preserved to ensure proper growth of the flap. The gastrocnemius muscle can then be rotated over the anterior aspect of the knee to cover a soft-tissue defect either via a subcutaneous tunnel or by dividing the intervening skin bridge.

##### Gracilis

The gracilis muscle is a long and thin unipennate muscle in the medial compartment of the thigh that originates on the medial aspect of the ischiopubic ramus and joins with the sartorius and semitendinosus muscle tendons to form the pes anserine [15]. It is primarily supplied by the medial circumflex femoral artery and branches of the superior femoral artery with innervation from the anterior branch of the obturator nerve [7].

Rao et al. described the operative approach for completing a gracilis muscle flap [7, 16, 17]. For this procedure, the patient is placed supine and the thigh is abducted. The gracilis muscle may be located by palpating 2–3 cm posterior from a line connecting the pubic symphysis to the medial condyle. The vascular supply to the gracilis muscle may be palpated and confirmed with Doppler ultrasonography 8–10 cm inferior to the pubic tubercle. Rao et al. recommend the use of a 10–15 cm sagittal incision from the pubis to the medial anterior knee to blunt dissect the gracilis from the sartorius and the semimembranosus muscles [7]. The vascular pedicles may be found running superficial to the adductor magnus muscle, underneath the adductor longus. When retracting the adductor longus muscle, the artery may be traced to its origins off the medial circumflex femoral artery. The muscle should then be released from its superior attachment site, reversed 180°, and tunneled under the skin to reach the defect over the knee.

#### Free flaps

##### Latissimus dorsi

The latissimus dorsi (LD) muscle is one of the most used free flaps for patients undergoing TKA with infected knees and/or extensive soft-tissue defects such as an exposed prosthesis, followed by the rectus abdominis muscle [7, 18, 19]. It may be taken as either a muscle or myocutaneous flap with sufficient bulk to cover large volumes of dead space [17]. A major disadvantage of using an LD flap is its high rate of donor site complications, which includes wound dehiscence, seroma, and functional morbidity limiting overhead activities [7, 18, 19].

When harvesting the LD flap, the patient is placed in a lateral decubitus position. An oblique incision is made along the anterior aspect of the muscle. The flap is elevated off the posterior thorax from its broad origin, which extends from the thoracic spinous processes and posterior iliac crest and is excised in cephalad direction toward the axilla at its humeral insertion. Segmental perforating vessels are ligated sequentially if necessary, while care is taken to protect the dominant pedicle, which is primarily supplied by the thoracodorsal artery [7, 18–22].

##### Rectus abdominis

The rectus abdominis serves as an excellent source for a free flap in the setting of revision knee arthroplasty as it provides a reliable anatomy with a large area for soft-tissue coverage when regional rotational flaps do not suffice [23]. It can be harvested with the patient supine with relatively low patient morbidity as a muscle-only, myofascial, or commonly as a myocutaneous flap without the need for any intraoperative position changes. When taken as a myocutaneous flap, the need for a separate split-thickness skin graft can be avoided depending on the size of the soft-tissue deficit.

The paired rectus abdominis muscles arise from the pubic symphysis and crest and insert onto the fifth, sixth, and seventh costal cartilages, providing truck flexion. The rectus abdominis is divided by fibrous tendinous intersections and is enclosed by an anterior and posterior sheath bound laterally by the linea semilunaris and medially by the linea alba. The investing rectus sheath is formed from contribution from the internal oblique, external oblique, and transverse abdominis fascia. The rectus abdominis is supplied by two dominant vascular pedicles: the deep superior and inferior epigastric vessels. The inferior epigastric artery, which enters the lateral border of the rectus muscle just beneath the arcuate line, is preferred for usage as a vascular pedicle [24–26].

During harvest, a longitudinal incision is made down to the level of the anterior rectus sheath with elevation of skin and subcutaneous tissue. The muscle can be freed from the posterior fascia and harvested separately when a muscle-only flap is used. Care is taken to identify and protect the inferior epigastric vessels as well as the integrity of the posterior sheath as the vessel runs deep to the muscle belly and superficial to the fascia.

#### Additional flap types

##### Fasciocutaneous

The use of local fasciocutaneous (FC) perforator flaps was first described in the leg by Pontén in 1981 and was later used by Lewis et al. to cover exposed knees [27, 28]. They consist of skin, subcutaneous tissue, and deep fascia, and offer great value in repairing soft-tissue defects around the knee with reduced functional morbidity at the donor site, resistance to infection, and an improved aesthetic result [18, 29]. The vascular supply comes from perforating arteries that enter the deep fascia either through an underlying muscle, within the intermuscular septum between adjacent muscles, or via a direct cutaneous branch [18, 29]. Commonly used FC flaps include the anterolateral thigh flap, sural artery flap, saphenous artery flap, genicular artery flaps, and anterior tibial artery flap [19].

##### Chimeric

Chimeric flaps are a more recent development in the realm of skin coverage for wound coverage. Chimeric flaps have been described for over 40 years, but their popularity has increased owing to their versatility as of late. Chimeric flaps are typically made up of at least one muscle perforator flap component and can be either perforator based or branch based [30]. Perforator-based chimeric flaps are composed of multiple independent flaps in which perforators arise from a common source vessel, whereas branch-based chimeric flaps are composed of multiple independent flaps that arise from independent subfascial branches of a common source vessel [30]. Because of this, there are often a number of different anatomical areas from which a chimeric flap may be derived [31–37]. However, chimeric flaps reported for TKA reconstruction have mainly used the gastrocnemius muscle–chimeric sural artery perforator flap or anterolateral thigh (ALT) perforator flap taken with vascularized fascia lata [30, 31, 37, 38].

The gastrocnemius muscle–chimeric sural artery perforator flap receives its source vessel from the medial or lateral sural artery and is taken with the medial or lateral gastrocnemius, respectively [30, 39]. To summarize, the medial and lateral sural arteries are the most common branches that arise from the popliteal artery. They traverse down their respective head of the gastrocnemius along the deep surface. Their course then pierces their respective muscle, as musculocutaneous perforators, continuing through the deep fascia and continuing to the subdermal plexus. The largest perforators, best for flap creation, are typically found in the distal half of the gastrocnemius muscles and more commonly come from the medial sural artery.

The chimeric flap utilizing a ALT perforator flap combined with fascia lata is prepared in the same fashion as described in an earlier section. In brief, the ALT is supplied by the lateral femoral circumflex artery (LCFA) and can be harvested as either an FC flap or as a myocutaneous flap, depending on the presence or absence of the septocutaneous perforator vessels that branch off the LCFA [40]. An additional step is taken to harvest a portion of the fascia lata as the ALT perforator flap is being harvested [31, 36].

## Results

A total of six flap types were identified for inclusion in this review: (1) gastrocnemius (*n* = 421), (2) rectus abdominis (*n* = 3), (3) latissimus dorsi (*n* = 41), (4) fasciocutaneous (*n* = 78), (5) chimeric (*n* = 4), and (6) gracilis (*n* = 9) (Table 1).

Healing rates across flap types ranged from ~ 70% to 100%: (1) gastrocnemius (*n* = 311, 73.8%), (2) rectus abdominis (*n* = 3, 100%), (3) latissimus dorsi (*n* = 27, 67%), (4) fasciocutaneous (*n* = 55, 70%), (5) chimeric (*n* = 4, 100%), and (6) gracilis (*n* = 8, 93%) (Table 1). There was no significant difference when comparing healing rates across flap types (*p* = 0.39).

Complication rates across flap types ranged from ~ 20% to 60%: (1) gastrocnemius (*n* = 252, 59.9%), (2) rectus abdominis (*n* = 0, 0%), (3) latissimus dorsi (*n* = 19, 46.3%), (4) fasciocutaneous (*n* = 15, 19.2%), (5) chimeric (*n* = 1, 25%), and (6) gracilis (*n* = 5, 55.6%) (Table 1). There was a significant difference when comparing complication rates across flap types (*p* < 0.0001), with a significant difference being noted between gastrocnemius (59.9%) and fasciocutaneous (19.2%) complication rates (*p* < 0.0001). All other comparisons between flap types by complication rate were not significantly different.

Healing and complication rates by study and follow-up times were recorded for each individual study (Tables 2–7).

## Discussion

In this review, gastrocnemius flaps were the most utilized in the setting of revision TKA. While there was a significant difference noted between the complication rates for gastrocnemius versus fasciocutaneous flaps (*p* < 0.0001), healing rates across flap types were not significantly different (*p* = 0.39). The significant difference in complication rates between gastrocnemius and FC flaps may be explained by gastrocnemius flaps being frequently utilized for large wound defects in clinically complex cases, whereas fasciocutaneous flaps may be reserved for smaller wound defects. These data suggest that all flap types are viable depending on the extent of the wound defect and demonstrate equivalent, moderate wound healing rates with a minimum 1-year follow-up.

### Pedicled/local flaps

#### Gastrocnemius

In this review, we noted that the gastrocnemius flap was the most utilized flap type (*n* = 421), with a healing rate of 73.8% and complication rate of 59.9% (Table 1, 2). Many studies have confirmed the benefit and safety of rotational gastrocnemius flaps in TKA revision, noting that they produce greater results than primary closure alone [5, 6, 41–61]. Busfield et al. noted a 100% return in extensor function in a cohort of patients (*n* = 9) who underwent gastrocnemius muscle flap coverage [6]. They determined that medial gastrocnemius flap reconstruction could provide a successful salvage therapy in the setting of failed extensor mechanism allograft or an alternative for patients with poor soft-tissue coverage, infection, or debilitating medical conditions. Corten et al. noted a satisfactory result in 92% of patients (*n* = 24) who underwent gastrocnemius muscle flap coverage of infected TKA at a mean follow-up of 4.5 years (range 1–10 years) [3].

Gastrocnemius flaps have also been reported to increase tissue delivery of antibiotics because of increased blood flow to surgical sites and its properties as a natural spacer and cover for prostheses [62]. Carlesimo et al. reported a clinical absence of reinfection postoperatively in TKA patients with rotation flap who underwent 6 weeks of antibiotic therapy (*n* = 8) [62]. When assessing long-term outcomes of gastrocnemius flap use in TKA, a study by Houdek et al. noted that, of 83 patients in whom a gastrocnemius flap had been used (*n* = 18 for primary procedure and *n* = 65 for revision procedure), the 10-year revision and amputation-free survival rates following gastrocnemius flap coverage were 68% and 79%, respectively.[63]

#### Gracilis

The gracilis muscle flap has been shown to be useful for knee resurfacing, especially in the treatment of suprapatellar defects, with negligible donor site morbidity [8, 16, 17, 64, 65]. Previous articles have demonstrated its utility as a substitute for or supplement to gastrocnemius muscle flaps in the treatment of suprapatellar defects with negligible donor site morbidity [16, 17]. Tiengo et al. studied the use of an extended reversed gracilis flap in three patients with severe soft-tissue defects following infected TKA with a mean follow-up of 23.3 months (range 22–24 months) and found that all three cases successfully healed without complication or evidence of infection at latest follow-up [17]. In another study, Jung et al. followed one patient, who previously underwent multiple TKA revisions due to recurrent infection, being treated with a reverse gracilis muscle flap and reported no complications during a 24-month follow-up period [22].

Last, Chim et al. demonstrated the role of the gracilis muscle as a supplementary flap in augmenting coverage provided by gastrocnemius muscle flaps in two patients with mid- and distal femur osteosarcoma, respectively [8]. Both flaps survived without complications with excellent wound healing, and their patients were able to ambulate independently in the postoperative period. The authors concluded that there is utility in using the gracilis muscle for additional coverage, especially in cases that necessitate a long femoral stem replacement.

These studies have produced findings in line with our current study results, which have shown a 93% healing rate with the use of the gracilis muscle flaps (*n* = 9). (Tables 1, 7).

### Free flaps

#### Latissimus dorsi

In this review, LD flaps (*n* = 41) had a healing rate of 67% and complication rate of 46.3% (Table 1). Common complications for this flap type included recurrent infection, wound dehiscence, and flap failure (Tables 1, 4).

The use of an LD flap offers a reliable and durable option for wounds requiring extensive soft-tissue coverage and does not cause any functional restrictions in the knee joint [7, 66]. Early studies on treating and prophylactic LD flaps revealed promising limb salvage and healing rates, although not without complication [20, 21, 67]. In an early case series performed by Markovich et al., 12 patients undergoing TKA with muscle flap coverage were studied [22]. Five patients received LD free flaps either for prophylactic soft-tissue coverage done before definitive reconstruction (*n* = 2) or for treating muscle flap for infected prostheses with deficient soft-tissue coverage (*n* = 3). The wound was revascularized successfully in all knees with satisfactory healing of the muscle flap and subsequent healing of the implant. One patient treated with a prophylactic LD flap experienced late infection, though the authors attributed this to the use of a large tibial allograft to cover an additional bony defect. Two patients with chronic knee infection treated with LD muscle flaps experienced recurrent infection and subsequent second reimplantation and resection arthroplasty, respectively. Ultimately, the authors concluded that success in patients treated with muscle flaps is dependent on the reason for and the timing of the muscle flap. Thus, treating muscle flaps used for chronic knee infections had the worst outcomes, whereas prophylactic muscle flaps used for soft-tissue coverage for knees with adherent skin and scar tissue achieved greater success.

In another study, Cetrulo et al. studied the use of free tissue transfer on 11 patients with complex wounds and exposed prostheses, which included 6 cases of LD muscle flaps [20]. In their review, all of their free flaps were successful. The authors achieved limb salvage in all of their patients and prosthesis salvage in all but one patient who had their prosthesis removed at an outside hospital followed by knee arthrodesis. Hierner et al. reported the use of LD myocutaneous free flaps in 14 patients with insufficient soft tissue for prophylactic coverage in TKA [21]. Primary wound healing was achieved in eight patients, while skin breakdown occurred in five patients requiring secondary skin grafting, and fasciocutaneous flap in one patient.. There were three late recurrences of infection, with ultimate removal of the knee prosthesis followed by conversion to arthrodesis. Overall, the authors concluded that free myocutaneous LD flap transfer made TKA prosthesis implantation possible, prevented postoperative knee stiffness, and had a low rate of severe complications in patients with a high risk of wound-healing problems.

Lastly, a recent study conducted by Raymond et al. reviewed 18 consecutive patients with multiply revised TKA and LD free flap reconstruction [66]. Median follow-up time for their patients was 49 months (range 18–110 months). At latest follow-up, they found that 14 of 18 patients (77.8%) had maintained their implant. Seven of those patients were infection-free, while another seven were on suppressive antibiotics with the implant in situ and four had above-knee amputations. They reported a 5-year revision-free implant survival of 75% and found low functional outcome scores at latest follow-up. Thus, the authors concluded that LD free flap was a viable option for limb salvage, but that functional outcomes can be poor and there may be a significant risk of ongoing infection and amputation.

#### Rectus abdominis

Overall, our review found a 100% success rate in three patients reviewed with no complications reported (Tables 1, 3). Browne et al. were one of the first to recommend the utilization of the rectus abdominis flap as a salvage procedure for compromised soft tissue associated with total knee arthroplasty [68]. Here, they reported nine patients undergoing muscle flap for salvage of chronically infected total knee arthroplasty that required a two-stage revision. The study included seven patients undergoing local gastrocnemius flaps and two patients undergoing free flap for large soft-tissue defects: one latissimus dorsi and one rectus abdominis. Browne et al. reported 100% clearance of infection with successful reimplantation at 6 and 12 weeks respectively for the latissimus dorsi and rectus abdominis flaps without complications. Five of seven patients (71.4%) undergoing gastrocnemius flaps had successful reimplantation with additional complications of skin graft loss and loss of distal segment of the gastrocnemius flap.

Markovich et al. also utilized the rectus abdominis free flap as a salvage procedure for compromised tissues associated with total knee arthroplasty [67]. Here they described 12 patients who underwent muscle flaps for different indications and treatments goals: (1) prophylactic soft-tissue coverage, done before definitive reconstruction; (2) muscle flaps for treating infected prostheses with deficient soft tissue; and (3) salvage muscle flaps for wound dehiscence in the immediate postoperative period. Two of the 12 patients underwent rectus abdominis free flaps, the first for prophylactic coverage and the second for salvage for wound dehiscence. They overall reported a 100.0% successful revascularization rate at 4.1 years follow-up. Both patients treated with rectus abdominis flaps had successful clearance of infection and healed with limb salvage and improvements in arc of motion as compared with preoperatively, without complications.

Cetrulo et al. reported on 11 patients who underwent free tissue transfer in the setting of exposed hardware and complex wounds after total knee arthroplasty [20]. Six of these 11 patients underwent free rectus abdominis transfer, with the remaining 5 undergoing latissimus dorsi transfers. Despite 3 of the 11 patients having failed previous local rotational flaps, Certulo et al. achieved a 100% success rate and 100% limb salvage rate. One latissimus flap required explant and primary fusion owing to advanced bone loss and osteolysis, resulting in an overall 91% prosthetic retainment rate. No functional outcome scores were reported, but all patients remained ambulatory postoperatively.

While the rectus abdominis free flaps are a great option with high success rates in both limb and prosthesis salvage, their indications are more limited to larger defects in the often multiply revised patient. As compared with the more convenient and accessible local rotational flaps, rectus abdominis free flaps have been less reported in the literature but appear to have high successful healing rates with low complications reported.

### Additional flap types

#### Fasciocutaneous

In this review, fasciocutaneous flaps had a healing rate of 70.0% and a complication rate of 19.2% (Table 1) Common causes of complications included amputation, arthrodesis, wound dehiscence, and revision procedures (Tables 1, 5).

Historically, FC flaps have been used to preserve cutaneous innervation and manage incisional skin necrosis after TKA in patients without underlying deep infection or large defects such as an exposed prosthesis [19, 56, 58, 61, 64, 69–72]. In a case series of 17 patients performed by Menderes et al. in which 7 TKA patients received a FC flap for wound coverage, the authors found modest success with limb salvage and complication rate [19]. Three of the seven patients required a revision procedure with a medial gastrocnemius myocutaneous flap in place of the failed FC flap. The secondary surgical procedures of these three patients would go on to heal without complication. Therefore, the authors concluded that FC flaps are preferred when the soft defect is small, without exposure of the prosthesis. Adam et al. retrospectively studied 25 TKA cases using 9 FC flaps and 16 muscle flaps over a mean follow-up time of 5.4 years (range 1–10 years) and found that all of their patients with FC flaps healed successfully compared with 10 out of 16 muscle flaps [5]. However, the authors noted that this may be attributed to the fact that they used FC flaps for small defects whereas muscle flaps were used for major wound dehiscence. Conversely, Vaienti et al. studied the use of island neurofasciocutaneous sural flaps in 15 patients with exposed TKA prostheses over a mean follow-up period of 18 months and reported great success in limb salvage [73]. All of their flaps survived, and only two cases of hematoma and one case of recurrent aseptic fistula occurred. Siim et al. reviewed 18 cases (10 local FC flaps) with an average follow-up of 7 years (range 1–17 years), and noted that FC flap use may result in successful outcomes with early reconstructive surgery in TKA patients with sufficient soft-tissue coverage; however, long-term outcomes are variable [74].

The use of unilateral or bilateral local FC flaps in a V–Y pattern has also been shown to reliably cover soft-tissue defects more than 2 cm wide following TKA and obviate the need for secondary skin grafting [72]. Misra and Niranjan conducted a retrospective series following 15 patients who used local FC flaps for patellar and peripatellar defects, with 8 of their 15 study patients receiving a V–Y flap design [75]. They found healing success in 100% of their patients with minimal complications as only one patient experienced wound dehiscence. All patients achieved a “good” outcome with a mean follow-up of 13 months (range 3–24 months).

#### Chimeric

Chimeric flaps were one of the least reported flap types in the setting of TKA revision. In this review, we noted a 100% (*n* = 4) healing rate for the gastrocnemius muscle–chimeric sural artery perforator flap and ALT perforator flap combined with fascia lata as reported by a few case studies (Tables 1, 6) [30, 31, 37, 38]. Hallock et al. studied two patients being treated with the gastrocnemius muscle–chimeric sural artery perforator flap for wound coverage, one of who underwent TKA revision with a medial gastrocnemius muscle and medial sural artery perforator flap [30]. They experienced a minor complication due to iatrogenic causes caused by their orthopedic service, which led to devascularization of the chimeric flap during reimplantation that necessitated revision with a lateral gastrocnemius muscle and lateral sural artery perforator. The flap went on to heal uneventfully 1 year out. Their case highlights the importance of interdisciplinary collaboration between orthopedic surgeons and plastic surgeons to optimize patient outcomes, especially those undergoing revision TKA requiring a flap for soft-tissue coverage.

Han et al. also reported the use of a medial gastrocnemius muscle and medial sural artery perforator flap in two patients undergoing TKA revision and did not observe any postoperative complications [37]. In another study, Cho et al. followed six patients, one of who received a chimeric flap consisting of medial gastrocnemius muscle component and a medial sural artery adipofascial component, for soft-tissue coverage due to infection following TKA. At 36-month follow-up, their patient had full range of motion and no gait disturbance [38]. Lastly, Fu et al. studied the use of the chimeric ALT perforator flap with fascia lata in one patient who had skin necrosis following TKA and found no complications after a 12-month follow-up period [31].

Overall, the lack of literature reporting these flaps suggests that chimeric flaps may not be utilized as frequently, possibly because of their extensive surgical exposure and lack of available data on long-term outcomes. However, as the small sample shows, chimeric flaps do have promise in the setting of TKA revision surgery, with minimal donor site morbidity and functional impairment.

## Study limitations

There are limitations to this review worth reporting. First, the decision-making process utilized by operating surgeons when deciding on which flap type to utilize for their patient cohorts was not readily known. Second, not all studies uniformly reported healing and complication rates, requiring authors to extrapolate this information from descriptive results, figures, and supplemental files within the studies; this limitation also prevented this review from performing a formal review process or aggregating data to conduct a meta-analysis.

## Conclusions

Gastrocnemius flaps are by far the most utilized flap type in the setting of revision TKA. Healing rates in this review did not vary significantly across flap types, which suggests that all flap types are viable depending on the extent of the wound defect and demonstrate equivalent, moderate wound healing rates with a minimum 1-year follow-up. The appropriate flap for coverage of soft-tissue defects in TKA revision is based on defect size/location, physician experience, and patient tolerance. We envision that multidisciplinary collaboration and proper patient follow-up will be key to exploring flap use in revision TKA procedures in the coming years.

## Data Availability

Not applicable. All studies included in this review are available online.

## Abbreviations

TKA: Total knee arthroplasty
LD: Latissimus dorsi
LFCA: Lateral femoral circumflex artery
FC: Fasciocutaneous
